# Supplementary material for: Mus musculus papillomavirus MmuPV1 resists restriction by human APOBEC3B
Source: J Virol. 2026 Feb 11;100(3):e01922-25. doi: 10.1128/jvi.01922-25 (PMC13011340; doi:10.1128/jvi.01922-25)
Supplement: Supplemental material — Figures S1 to S5; Table S1. [file jvi.01922-25-s0001.pdf]

Supplementary Information for:

Mus Musculus papillomavirus MmuPV1 resists restriction by human APOBEC3B

Xingyu Liu[1], Andrea Bilger[2], Denis Lee[2], Prokopios P. Argyris[3], Jiarui Chen[4,5], Ella Ward-Shaw[2], Emilia Barreto Duran[1], Yu-Hsiu T. Lin[1], Cameron Durfee[1], Sang Hyun Chun[1], Mahmoud Ibrahim[1], Joshua Proehl[1], Allen J. York[1,6], Paul F. Lambert[2,#] and Reuben S. Harris[1,6,#]

[1] Department of Biochemistry and Structural Biology, University of Texas at San Antonio, San Antonio, Texas, USA

[2] McArdle Laboratory for Cancer Research, School of Medicine and Public Health, University of Wisconsin-Madison, Madison, Wisconsin, USA

[3] Department of Pathology, University of Chicago Medicine, Chicago, Illinois, USA

[4] Division of Surgical Oncology, Department of Surgery, School of Medicine and Public Health, University of Wisconsin-Madison, Madison, Wisconsin, USA

[5] Center for Precision Medicine, University of Wisconsin-Madison, Madison, Wisconsin, USA

[6] Howard Hughes Medical Institute, University of Texas at San Antonio, San Antonio, Texas, USA

# Correspondence: RSH rsh@uthscsa.edu; PFL plambert@wisc.edu

Running Title: MmuPV1 resists A3B restriction

Supplement Information: Supplementary Figures S1-S5 and Table S1

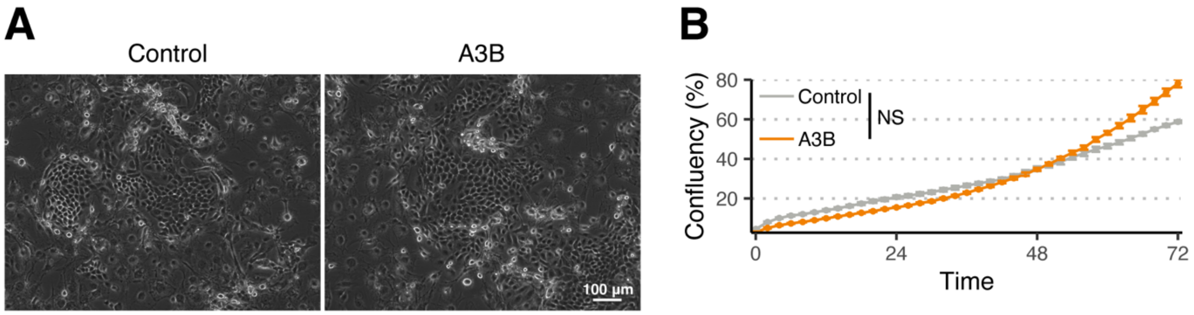

FIG S1 Morphology and proliferation curve between A3B-expressing and control cells. (**A**) Bright-field images with 10X magnification acquired from either control (rep1 MKB051) or A3B-expressing keratinocytes (rep1 MKB056) co-cultured with a feeder layer. Polygonal-shaped cells (keratinocytes) are supported by surrounding spindle-shaped cells (feeder layer).

(**B**) Cell proliferation curve for control (rep1 MKB051) and A3B-expressing keratinocytes (rep2 MKB056). Bright-field images were acquired and confluency rates enumerated (mean +/- SD from n = 6 technical replicates; $p = 0.99$ by two-factor, two-way ANOVA).

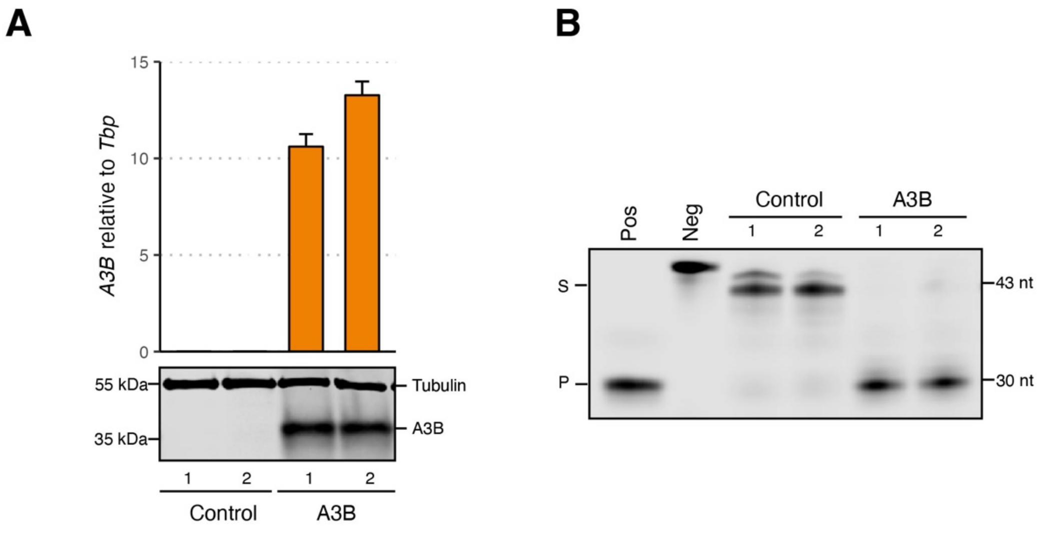

FIG S2 Independent validation of murine keratinocytes.

(**A**) RT-qPCR and immunoblot results of human A3B transcripts and protein expression from an independent pair of two control (rep3 MKB070, rep4 MKB071) and two A3B-expressing keratinocytes (rep3 MKB067, rep4 MKB069).

(**B**) DNA deamination activity of extracts from indicated murine keratinocyte cell cultures (S: substrate; P: products). Negative control: substrate with HED buffer. Positive control: substrate with HED buffer containing recombinant A3A.

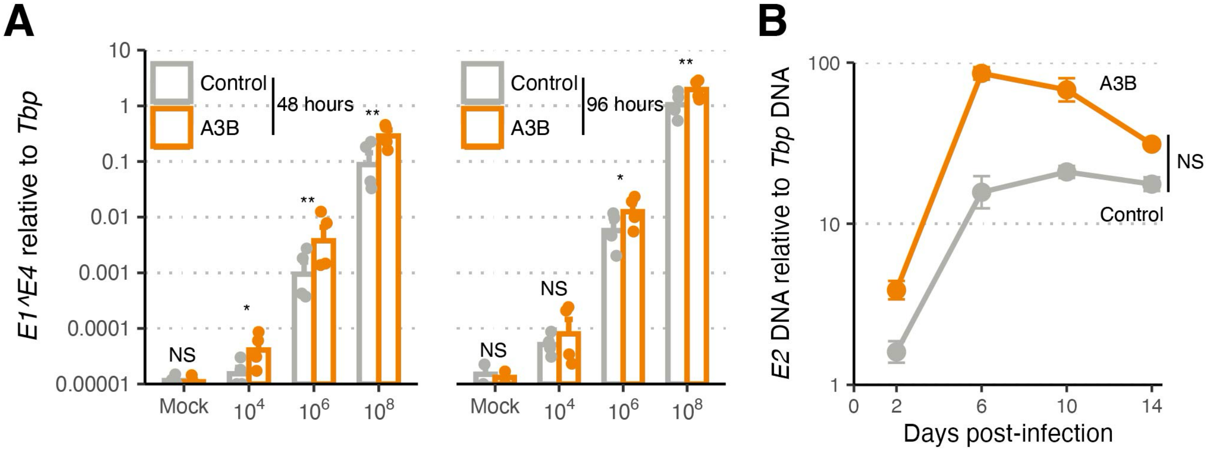

FIG S3 Infection quantification of validation cells.

(**A**) RT-qPCR results of MmuPV1 E1^E4 splicing transcripts levels relative to *Tbp* mRNA from cell extracts 48 hours or 96 hours p.i., respectively. Data from two control (rep3 MKB070, rep4 MKB071) and two A3B groups (rep3 MKB067, rep4 MKB069) were combined [n = 3 infections per group with individual data points and means shown; *, $p < 0.05$; **, $p < 0.01$; ***, $p < 0.001$; and $p \geq 0.05$, non-significant (NS) by Mann-Whitney U test].

(**B**) qPCR results of MmuPV1 E2 DNA copies relative to *Tbp* genomic DNA (mean +/- SD from n = 2 independent experiments, each with 2 cultures; $p = 0.08$ by two-factor, two-way ANOVA)

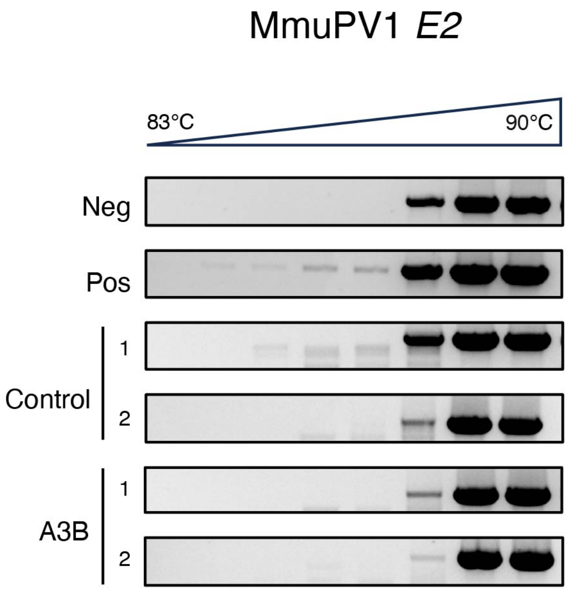

FIG S4 Differential DNA denaturation PCR (3D-PCR) of MmuPV1 *E2* region from two control and two A3B-expressing keratinocytes day 14 DNA extracts. Negative control: pMmuPV1; Positive control: pMmuPV1 co-incubated with recombinant A3A.

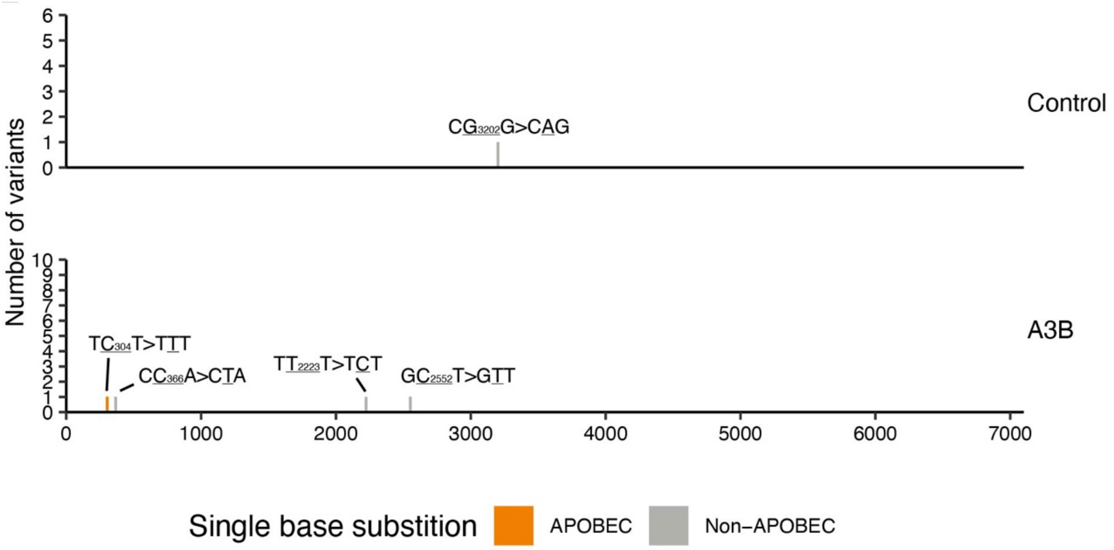

FIG S5 MmuPV1 genome-wide variants distribution. DNA extracts at day 14 infection from control rep2 and A3B rep1 were amplified and PCR-cloned. A total of 6 and 10 clones from control rep2 or A3B rep1 were sequenced (orange: APOBEC-preferred context; grey: other contexts).

Table S1 Primers used in manuscript

Category	Gene	Sequences (5'→3') Forward	Sequences (5'→3') Reverse
Genotyping	WT Rosa26	AGCACTTGCTCTCCCAAAGTC	CACCTGTTCAATTCCCCTGC
	LSL-A3Bi	GAAACATAAAATGAATGCAATTGTTGTTG	TCCGCTCCATCGGATTTCTG
	L-A3B	CGTGCTGGTTATTGTGCTGT	TCCGCTCCATCGGATTTCTG
RT-qPCR	E1^E4	CATTCGAGTCACTGCTTCTGC	GATGCAGGTTTGTCGTTCTCC
	E1^E4 probe	6-carboxyfluorescein (FAM)-TGGAAAACGATAAAGCTCCTCCTCAGCC-6-carboxytetramethylrhodamine (TAMRA)	
	Tbp	GGGGAGCTGTGATGTGAAGT	CCAGGAAATAATTCTGGCTCA
	A3B	GACCCTTTGGTCCTTCGAC	GCACAGCCCCAGGAGAAG
qPCR	E2	GCCCGAAGACAACACCGCCACG	CCTCCGCCTCGTCCCCAAATGG
	Tbp	GGAGAGGGCTGTATGTGTATT	CAGAAGGCAGAGACAGAATGAG
3D-PCR	E2	ATGGGCAGCCAGTATATCCC	ACTGTTTACTGGGGGCTTACTG
	LCR	TAACAGCTAATGGGCAGCCA	CCGTTCCCGCTTCACCTAAA
Whole viral genome	WG	GTCAAGCACAATGTCCTCAAA	AGTGGTACTGAAGAGCTGATT
